# Genetic Variation in DNA Repair Pathways and Risk of Non-Hodgkin's Lymphoma

**DOI:** 10.1371/journal.pone.0101685

**Published:** 2014-07-10

**Authors:** Justin Rendleman, Yevgeniy Antipin, Boris Reva, Christina Adaniel, Jennifer A. Przybylo, Ana Dutra-Clarke, Nichole Hansen, Adriana Heguy, Kety Huberman, Laetitia Borsu, Ora Paltiel, Dina Ben-Yehuda, Jennifer R. Brown, Arnold S. Freedman, Chris Sander, Andrew Zelenetz, Robert J. Klein, Yongzhao Shao, Mortimer Lacher, Joseph Vijai, Kenneth Offit, Tomas Kirchhoff

**Affiliations:** 1 NYU School of Medicine, New York University, New York, New York, United States of America; 2 Memorial Sloan-Kettering Cancer Center, New York, New York, United States of America; 3 Hadassah-Hebrew University Medical Center, Jerusalem, Israel; 4 Dana Farber Cancer Center, Harvard University, Boston, Massachusetts, United States of America; IRCCS National Cancer Institute, Italy

## Abstract

Molecular and genetic evidence suggests that DNA repair pathways may contribute to lymphoma susceptibility. Several studies have examined the association of DNA repair genes with lymphoma risk, but the findings from these reports have been inconsistent. Here we provide the results of a focused analysis of genetic variation in DNA repair genes and their association with the risk of non-Hodgkin's lymphoma (NHL). With a population of 1,297 NHL cases and 1,946 controls, we have performed a two-stage case/control association analysis of 446 single nucleotide polymorphisms (SNPs) tagging the genetic variation in 81 DNA repair genes. We found the most significant association with NHL risk in the *ATM* locus for rs227060 (OR = 1.27, 95% CI: 1.13–1.43, p = 6.77×10^−5^), which remained significant after adjustment for multiple testing. In a subtype-specific analysis, associations were also observed for the *ATM* locus among both diffuse large B-cell lymphomas (DLBCL) and small lymphocytic lymphomas (SLL), however there was no association observed among follicular lymphomas (FL). In addition, our study provides suggestive evidence of an interaction between SNPs in *MRE11A* and *NBS1* associated with NHL risk (OR = 0.51, 95% CI: 0.34–0.77, p = 0.0002). Finally, an imputation analysis using the 1,000 Genomes Project data combined with a functional prediction analysis revealed the presence of biologically relevant variants that correlate with the observed association signals. While the findings generated here warrant independent validation, the results of our large study suggest that *ATM* may be a novel locus associated with the risk of multiple subtypes of NHL.

## Introduction

The incidence of non-Hodgkin's lymphoma (NHL) in the U.S. has doubled over the past two decades. While the etiology of the disease remains largely unknown [1], surmounting evidence suggests that genetic predisposition plays a role in NHL development [2]–[4]. Besides recently completed genome-wide association studies (GWAS) [5]–[13], the search for missing genetic susceptibility to lymphoma in the past decade also involved the association analyses of common genetic variants in candidate molecular pathways putatively involved in lymphoma development. In contrast to GWAS, candidate scans allow for the focused assessment of biologically relevant molecular pathways by testing larger sample populations and maintaining a higher statistical power for detecting association effects [14]. Among the candidate networks previously investigated for the association with NHL risk, DNA repair was frequently explored [15]–[25] due to its strong relevance to lymphomagenesis [26]–[28].

Associations between DNA repair genes and lymphoma risk have been reported previously [15]–[19], however the results in many of these studies either failed to reach the necessary level of statistical significance, or lacked independent validation. In the present study, we attempted to improve on these prior efforts by performing a two-stage case-control analysis of 1,297 NHL cases and 1,946 controls to identify associations between 446 SNPs tagging 81 DNA repair genes and NHL risk. The two-stage design, thorough selection of DNA repair genes, assessment of genetic interactions, and identification of putatively functional variants by using public genomic and expression data are among the major innovations in our study, which provides yet another focused exploration of the role of DNA repair pathways in genetic susceptibility to NHL.

## Materials and Methods

### Ethics Statement

All cases were ascertained through Memorial Sloan-Kettering Cancer Center (MSKCC) IRB-approved protocols, or a protocol approved by the IRB at the Dana Farber Cancer Institute (DFCI) or Hadassah-Hebrew University. These protocols required written informed consent either for identified use of specimens for research into the genetic basis of lymphoma, or research use of specimens permanently de-identified prior to genotyping. Controls were part of the New York Cancer Project (NYCP) and all subjects gave written consent for use of samples in genetic studies of any disease state.

### Study population

In total, the study involved 1,297 non-Hodgkin's lymphoma (NHL) cases from the combined resources at MSKCC, DFCI and Hadassah-Hebrew University, Israel as well as 1,946 controls collected from the NYCP, a study of 18,000 New York City residents originally designed to assess the role of environment and genetics in cancer risk, and described previously elsewhere [29]–[31]. The NYCP data include age, gender, history of cancers (including lymphoma) and ethnicity. A subset of NHL cases (n = 222) were probands from families with a strong family history (FH) of NHL, described in detail recently [32]. The remaining fraction of NHL patients (n = 1,075), were unrelated and unselected for FH. All cases and controls were of white European ancestry, with a fraction of cases (n = 534, 41.2%) and controls (n = 1,043, 53.6%) of self reported Ashkenazi Jewish (AJ) ancestry. In this study we have employed a two-stage design; the discovery stage (stage 1) consisting of 650 cases and 965 controls, and the replication stage (stage 2) involving 647 cases and 981 controls. The detailed structure, demographic, and clinical information of case/control populations in both stage 1 and 2 is summarized in Table S1. A subset of the patient collection in this study was previously included in a GWAS on lymphoma susceptibility [9]. Of the 944 lymphoma cases that constituted the GWAS phase of that prior study, 515 cases (39.7%) overlap with the 1,297 patients included here. While 1,043 controls (53.6%) overlap between the current study and the validation stage of the prior GWAS, there was no control overlap between this study and the GWAS discovery stage.

### Selection of genes and tagging SNPs (tSNPs)

The selection of candidate DNA repair genes was performed as summarized in Figure S1. The initial subset of genes (n = 34) has been identified for their known role in DNA repair processes and queried for their catalytic activities from Gene Ontology (GO) [33] and KEGG [34]. The key networks of DNA repair defined by the catalytic domains in the seed list were further passed to: 1) GO search for genes containing identified catalytic activities and 2) yeast proteome database [35] identifying yeast homologues with experimental evidence demonstrating their effect on UV sensitivity, radiation, and DNA damage response. The yeast genes were subsequently queried for human homologues. The targets from 1) and 2) were crossed for gene overlap and passed to an interactome analysis (GeneGO, Ingenuity) to query interacting partners (defined by at least two independent reports, and confirmed by at least two experimental methods). After merging, 87 DNA repair genes were identified for the study (Table S2). The SNPs tagging 87 selected genes were chosen using Haploview with a haplotype Pearson's correlation coefficient (r^2^) threshold <0.6 across selected gene regions (including 5 kb from 3′ and 5′ UTR), and minor allele frequency (MAF) >0.05. The tagging SNP selection has been performed using the CEU data (120 individuals) of HapMap Phase II, the most accurate resource available for this purpose at the time of the study design and still serves as the most validated reference for capturing the common genetic variation in European populations. In total, 531 tagging SNPs (tSNPs) were selected to tag the 87 DNA repair genes.

### Genotyping

For stage 1, the genotyping of 531 tSNPs from 87 selected DNA repair genes on 698 lymphoma cases and 1,041 controls was conducted using Sequenom MassARRAY iPLEX (Sequenom Inc., CA), multiplexed into a 16-plex design as per manufacturer's protocol and as described previously [30]. For quality control (QC), duplicates (8 per each 384-well plate) showed >99% concordance and non-template controls (2 per plate) revealed no evidence of cross-contamination. Thirty-four SNPs were excluded due to low genotyping rate across samples (<85%), poor clustering, or significant departure from Hardy-Weinberg Equilibrium (p<0.001 in control population); an additional 51 SNPs were dropped due to low MAF (<0.05) in our study population. Forty-eight cases and 76 controls were dropped due to low genotyping rate (<85%). After QC, in total 446 SNPs, tagging 81 DNA repair genes and n = 650 cases and n = 965 controls remained for the association analysis in stage 1. Twenty-eight SNPs associated with NHL (p<0.05) from stage 1 were passed to the validation analysis in stage 2 on an additional 684 cases and 1,042 controls. In order to perform downstream haplotype and imputation analyses, in stage 2 we also included 81 additional SNPs tagging the genes captured by the 28 significant loci in stage 1. A total of 109 SNPs were passed to the replication analysis in stage 2 using a re-plexed Sequenom design (iPLEX). While all 109 SNPs passed the QC in stage 2, 37 cases and 61 controls were dropped due to low genotyping rates across SNPs (<85%), resulting in the genotyping data on 109 SNPs in 647 cases and 981 controls. Data can be made available to other researchers: please contact the authors for details.

### Statistical association analyses

Single SNP associations with NHL risk were tested using a logistic regression model under a per-allele test, calculating odds ratios (OR) and 95% confidence intervals (95% CI), adjusted for age, gender and AJ status. The associations were analyzed for stage 1, stage 2, and the aggregate sample set including both stages. In the aggregate sample set we also used a per-allele logistic regression model to test fifteen SNP associations within the three most common NHL subtypes in our dataset: diffuse large B-cell lymphoma (DLBCL), follicular lymphoma (FL) and small lymphocytic lymphoma/chronic lymphocytic leukemia (SLL/CLL). Quantile/quantile (Q/Q) plots were produced using *ggplot2* in R, and inflation factors (λ) were calculated based on 90% of least significant SNPs. Statistical analyses were performed using PLINK [36]. The main analysis examining single SNP associations with NHL risk and the associations among subtypes were controlled for multiple testing using Bonferroni adjustment. The Bonferroni level of significance was defined as p<0.000154, accounting for 247 independent SNPs in stage 1, 33 independent SNPs in stage 2, and 15 SNPs tested among three NHL subtypes (number of tests = 247+33+(15*3) = 325; p = 0.05/325 = 0.000154). Independent SNPs were defined under Pearson's correlation coefficient (r-square) <0.5 calculated among our sample population.

Haplotypes were visualized by Haploview and haplotype associations were performed by logistic regression analysis adjusted for age, gender, and AJ status. We have also tested the homogeneity of the odds ratios between these three major subtypes by calculating the Breslow-Day statistics for each SNP.

In order to explore possible epistatic interactions associated with NHL risk, logistic regressions were modeled by adding an interaction term between the genotypes of each SNP pair. First, pairwise comparisons were performed between each SNP from the list of fifteen associations with NHL (105 tests), followed by pairwise comparisons of fifteen SNPs with the additional 94 SNPs included in the aggregate analysis (1,410 tests, Bonferroni adjusted p-value: 0.05/(105+1410)  = 3.3×10^−5^). This analysis was performed using PLINK [36]. For the targeted analysis of the MRN complex (*MRE11A*, *NBS1*), the multiplicative two-way gene-gene interactions were estimated using multiple logistic regression models. For each SNP pair, a logistic regression model was built to test case/control association based on the indicator variables (sex, age and AJ status) and the 2-SNP variable, for a total of 5 variables and an intercept. The 2-SNP variable was defined separately under three genetic models, based on the number of risk alleles in individual subjects.

For the assessment of SNP-gene association by incorporating expression information (expressed quantitative trait loci – eQTL) we used Genevar [37]. The eQTL associations were calculated by Spearman's rank correlation tests.

### Imputation and functional prediction

The imputation of genotypes from 1,297 NHL cases and 1,946 controls was performed using IMPUTE2 [38] with a reference panel consisting of the 1,000 Genomes Project (1KG) data freeze from November 2010 for low-coverage genomes, May 2011 for high-coverage exomes, and the phased haplotypes released March 2012 (n = 1,092 individuals). Imputed loci were filtered based on the quality metric score (info score) >0.7 [39], chosen based on inflation estimates from a Q/Q analysis; the imputed SNPs with info score <0.7 showed significant inflations in our data (Figure S2). The functional annotation of the associated tagging SNPs and their correlated imputed SNPs was performed by ANNOVAR [40], focusing on 8 functional categories: coding regions, conserved transcription factor (TF) binding sites, TF binding sites based on ChIP-Seq data (using ENCODE database), enhancer sites based on H3K4me1 chromatin marks (using ENCODE database), DNase I hypersensitivity clusters (using ENCODE database), known CNVs, and 3′ UTR, and 5′ UTR.

## Results

### Sample population

The demographic and clinical composition of our sample population is summarized in Table S1. No significant difference was observed in the distribution of demographic variables or lymphoma subtypes between stage 1 and stage 2. Also, no significant difference between demographic characteristics or subtype distribution has been noted between cases with FH and NHL cases unselected for FH. However, there was a difference in the proportion of age, AJ ancestry and gender between the NHL cases and controls. Hence, age, AJ status and gender were used as covariates in all subsequent statistical analyses.

### Single SNP associations with NHL risk

In this study we applied a two-stage design: in stage 1 we performed the association analysis on 446 SNPs tagging 81 DNA repair genes in the population of 650 cases and 965 controls. There was no significant inflation in observed versus expected associations (λ = 1.07), indicating no detectable genotyping artifacts or population substructures impacting our findings (Figure S3).

We first tested the association of DNA repair variants with NHL cases (all subtypes pooled). The association analysis of NHL cases in stage 1 identified 28 SNPs associated with NHL risk (p<0.05). The strongest associations in stage 1 were found for 3 SNPs in the *ATM* locus: rs611646, rs419716 and rs227060, the latter showing the strongest effect (OR = 1.27, 95% CI: 1.07–1.49, p = 0.005). Other associations in stage 1 included tSNPs in *MRE11A* (rs625245, OR = 0.78, 95% CI: 0.65–0.93, p = 0.006), *GTF2H1* (rs4150606, OR = 0.82, 95% CI: 0.69–0.97, p = 0.02), and *MSH2* (rs4952887, OR = 0.66, 95% CI: 0.47–0.91, p = 0.01).

The twenty-eight most significant SNPs from stage 1 and an additional 81 SNPs, as described in Materials and Methods (total 109 SNPs) were passed to stage 2, which involved 647 NHL cases and 981 controls. The associations were replicated for rs227060 and rs611646 in *ATM* with a more pronounced effect than in stage 1; rs227060 again shows the strongest association in stage 2 (OR = 1.30, 95% CI: 1.10–1.54, p = 0.002). Other SNPs that replicated in stage 2 include *GTF2H1* (rs4150606, OR = 0.81, 95% CI: 0.68–0.95, p = 0.01), *MSH2* (rs4952887, OR = 0.76, 95% CI: 0.58–1.01, p = 0.05) and *MRE11A* (rs625245, OR = 0.84, 95% CI: 0.69–1.01, p = 0.05). See Table S3 for the complete association results.

In the aggregate analysis of stage 1 and 2, fifteen SNPs showed associations with NHL risk (Table 1). These included two SNPs in the *ATM* locus: rs227060 and rs611646 (OR = 1.27, 95% CI: 1.13–1.43, p_agg_ = 0.00007; OR = 1.26, 95% CI: 1.12–1.43, p_agg_ = 0.00015, respectively, where p_agg_ is a p-value from aggregate analysis). Importantly, the associations for both loci remained significant after Bonferroni correction, as detailed in Material and Methods (p_adj_  = 0.022; p_adj_ = 0.049, respectively, where p_adj_ is the Bonferroni corrected p-value for each SNP). The two SNPs show incomplete LD (r^2^ = 0.642, Figure S4). In order to test whether the associations in *ATM* were independent we conditioned the analyses by the status of rs227060 and found rs611646 and rs419716 no longer significant (data not shown), suggesting the association signals observed for *ATM* SNPs are correlated. No other SNP associations in the aggregate analysis passed Bonferroni correction. To further explore the structure of associated loci, we also examined common haplotypes (MAF>0.05) for association with NHL risk. The strongest risk effect has been observed for a haplotype in *ATM*. Other loci have also shown specific haplotypes associated with NHL risk, however none were more significant than the associations from single SNP analyses (Table S4). As our study population includes a large fraction of AJ ancestry, we have investigated potential association differences between AJ and non-AJ samples. The majority of the 109 SNPs (n = 94) in the aggregate analysis show no more than a 5% difference in minor allele frequency (MAFs) between AJ and non-AJ subsets (Figure S5). Three SNPs (rs4150606, rs7149962, rs7562048) that present with >5% difference in MAF also show association with NHL. The analysis stratified by AJ status indicated that most associations, including our two most significant *ATM* SNPs, appear to be largely driven by non-AJ subsets which are predominant in our case population (Table S5). However, for other SNP associations such as rs4150606 (*GTF2H1*), rs4952887 (*MSH2*), and rs702019 (*POLQ*), both AJ and non-AJ subsets contribute to the association signal. Therefore, all analyses in the study were also adjusted by AJ status.

**Table 1:** Association analysis of genetic variants in DNA repair genes with the risk of NHL.

					Stage 1		Stage 2		Stage 1 & 2 [Aggregate]	
					(650 Cases/965 Controls)		(647 Cases/981 Controls)		(1,297 Cases/1,946 Controls)	
Gene	Chr.	SNP	Minor Allele	Allele Freq.	OR (95% CI)	*P*	OR (95% CI)	*P*	OR (95% CI)	*P*
ATM	11	rs227060	T	0.37	1.27 (1.07–1.49)	0.0057	1.30 (1.10–1.54)	0.0020	1.27 (1.13–1.43)	6.77×10^−5^
ATM	11	rs611646	T	0.45	1.24 (1.04–1.49)	0.016	1.30 (1.10–1.54)	0.0020	1.26 (1.12–1.43)	1.52×10^−4^
GTF2H1	11	rs4150606	A	0.50	0.82 (0.69–0.97)	0.021	0.81 (0.68–0.95)	0.013	0.81 (0.72–0.92)	6.68×10^−4^
MSH2	2	rs4952887	T	0.07	0.66 (0.47–0.91)	0.012	0.76 (0.58–1.01)	0.055	0.71 (0.57–0.87)	0.00128
MRE11A	11	rs625245	C	0.32	0.78 (0.65–0.93)	0.0066	0.84 (0.69–1.01)	0.059	0.81 (0.71–0.93)	0.0018
ATM	11	rs419716	T	0.42	0.8 (0.67–0.96)	0.014	0.85 (0.71–1.01)	0.056	0.83 (0.73–0.94)	0.0025
POLQ	3	rs702019	G	0.23	0.77 (0.62–0.95)	0.013	0.90 (0.74–1.09)	0.271	0.84 (0.73–0.96)	0.013
TDP1	14	rs7149962	C	0.06	1.49 (1.07–2.08)	0.020	1.19 (0.85–1.65)	0.307	1.33 (1.05–1.68)	0.019
NBS1	8	rs1805812	C	0.06	0.87 (0.59–1.27)	0.466	0.65 (0.46–0.94)	0.021	0.75 (0.58–0.97)	0.028
CCNH	5	rs3093819	T	0.46	1.14 (0.96–1.34)	0.128	1.13 (0.96–1.34)	0.139	1.14 (1.01–1.28)	0.030
MRE11A	11	rs10831227	A	0.34	0.93 (0.79–1.1)	0.382	0.83 (0.70–0.99)	0.035	0.88 (0.78–0.99)	0.030
CHEK1	11	rs565416	A	0.32	0.79 (0.66–0.94)	0.0075	0.99 (0.84–1.17)	0.892	0.88 (0.78–0.99)	0.037
MSH6	2	rs7562048	G	0.44	0.79 (0.66–0.93)	0.0062	0.98 (0.82–1.16)	0.818	0.88 (0.78–0.99)	0.039
NBS1	8	rs14448	C	0.06	0.99 (0.70–1.40)	0.936	1.61 (1.16–2.23)	0.0043	1.28 (1.01–1.62)	0.041
ATR	3	rs10804682	A	0.20	1.24 (1.02–1.51)	0.031	1.09 (0.90–1.32)	0.375	1.15 (1.01–1.32)	0.042

OR = odds ratio; CI = confidence interval.

### The associations of DNA repair genes with NHL subtypes

We have further tested the associations identified in the NHL pooled analysis among NHL subtypes on fifteen SNPs that associated with overall NHL risk in the aggregate analysis. We focused on the three most common NHL subtypes in our study population in order to maintain analytical power: diffuse large B-cell lymphomas (DLBCL), follicular lymphomas (FL), and small lymphocytic lymphoma/chronic lymphocytic leukemia (SLL/CLL). As shown in Table 2, associations were identified for the three *ATM* tSNPs in DLBCL (n = 412), with the strongest effect for rs611646 (OR = 1.37, 95% CI 1.14–1.64, p = 0.0008). Associations for rs611646 and rs227060 were also observed in SLL/CLL (n = 164). No *ATM* tSNPs were associated with risk in the FL subset (n = 301). In contrast, the strongest effects in FL was observed for *CHEK1* (rs565416, OR = 0.71, 95% CI: 0.58–0.87, p = 0.001) and *TDP1* (rs7149962, OR = 1.64, 95% CI: 1.14–2.35, p = 0.007), which were not associated with DLBCL or SLL/CLL. In SLL/CLL, the strongest association effect was found for rs4150606 tagging *GTF2H1* (OR = 0.51, 95% CI: 0.39–0.69, p = 4.84×10^−6^); this association was not seen in FL or DLBCL sub-analyses. The association of rs4150606 with SLL/CLL remains significant after Bonferroni correction for multiple testing (p_adj_ = 0.0016). The SNP/MAF plot between AJ and non-AJ (Figure S5) identified rs4150606 as an outlier. Despite the MAF difference it appears that both AJ and non-AJ ancestries contribute to the observed association risk effect of rs4150606 (Table S5).

**Table 2:** Association analysis of genetic variants in DNA repair genes with the risk of major NHL subtypes.

						DLBCL		FL		SLL/CLL		
						(412 Cases/1,946 Controls)		(301 Cases/1,946 Controls)		(164 Cases/1,946 Controls)		
Gene	Chr.	SNP	Minor Allele	Allele Freq.		OR (95% CI)	*P*	OR (95% CI)	*P*	OR (95% CI)	*P*	Breslow-Day *P*
ATM	11	rs611646	T	0.45		1.37 (1.14–1.64)	7.7×10^−4^	1.19 (0.96–1.46)	0.108	1.41 (1.06–1.87)	0.017	0.132
ATM	11	rs227060	T	0.37		1.34 (1.13–1.58)	8.7×10^−4^	1.10 (0.91–1.34)	0.334	1.58 (1.21–2.06)	6.7×10^−4^	0.037
ATM	11	rs419716	T	0.42		0.75 (0.62–0.90)	0.0026	0.84 (0.68–1.04)	0.107	0.84 (0.63–1.11)	0.217	0.229
MRE11A	11	rs10831227	A	0.34		0.77 (0.65–0.93)	0.0050	0.91 (0.75–1.11)	0.361	0.97 (0.74–1.26)	0.803	0.479
ATR	3	rs10804682	A	0.20		1.27 (1.05–1.55)	0.016	1.09 (0.86–1.37)	0.480	1.17 (0.87–1.59)	0.296	0.152
POLQ	3	rs702019	G	0.23		0.79 (0.64–0.97)	0.025	0.81 (0.64–1.03)	0.082	0.98 (0.71–1.34)	0.876	0.362
NBS1	8	rs14448	C	0.06		1.39 (1.01–1.93)	0.047	1.11 (0.74–1.65)	0.625	0.87 (0.48–1.58)	0.654	0.320
MSH2	2	rs4952887	T	0.07		0.72 (0.52–1.00)	0.048	0.71 (0.49–1.03)	0.073	0.63 (0.38–1.06)	0.079	0.432
CCNH	5	rs3093819	T	0.46		1.15 (0.98–1.37)	0.095	1.29 (1.06–1.57)	0.012	1.14 (0.87–1.49)	0.341	0.773
MRE11A	11	rs625245	C	0.32		0.85 (0.71–1.03)	0.106	0.78 (0.63–0.97)	0.028	0.87 (0.65–1.16)	0.347	0.810
GTF2H1	11	rs4150606	A	0.50		0.88 (0.74–1.05)	0.144	0.84 (0.69–1.02)	0.083	0.52 (0.39–0.69)	4.8×10^−6^	0.001
CHEK1	11	rs565416	A	0.32		0.97 (0.82–1.16)	0.749	0.71 (0.58–0.87)	0.0012	0.99 (0.76–1.28)	0.915	0.006
TDP1	14	rs7149962	C	0.06		1.06 (0.73–1.52)	0.773	1.64 (1.14–2.35)	0.0076	0.91 (0.52–1.60)	0.754	0.921
NBS1	8	rs1805812	C	0.06		0.68 (0.45–1.04)	0.073	0.72 (0.46–1.15)	0.168	1.05 (0.58–1.88)	0.877	0.831
MSH6	2	rs7562048	G	0.44		0.87 (0.73–1.05)	0.146	0.87 (0.71–1.06)	0.162	0.84 (0.64–1.09)	0.188	0.150

DLBCL = diffuse large B-cell lymphoma; FL = follicular lymphoma; SLL/CLL = small lymphocytic lymphoma/chronic lymphocytic leukemia; OR = odds ratio; CI = confidence interval.

We have also tested the association heterogeneity among the three major subtypes in our analysis of fifteen SNPs (Table 2). The Breslow-Day test results showed an association for rs227060 (p = 0.037), indicating heterogeneity in the odds ratios between the DLBCL, SLL/CLL, and FL. Heterogeneity has also been observed for an additional two SNPs with subtype-specific associations: rs4150606 (*GTF2H1*, p = 0.001) and rs565416 (*CHEK1*, p = 0.006).

### The epistatic SNP-SNP interactions in DNA repair genes and NHL risk

Using the additive model in pairwise SNP-SNP interaction analysis, we found several associations with NHL risk among the top fifteen SNPs from the aggregate analysis. However, none of these associations survived the adjustment for multiple testing (Table S6). Nonetheless, we noted several interactions of variants in *MRE11A* with SNPs in *NBS1* associated with NHL risk. Interestingly, both genes biologically interact in the MRN complex, a centerpiece of double strand break repair machinery, which prompted us to examine these interactions more closely using different genetic models. While all interactions involve rs1805812 in *NBS1*, for *MRE11A* there are four different variants which contribute to these associations (rs10831227, rs625245, rs607974, rs557148). After examination of the four pairwise associations (rs1805812 in *NBS1* with each of the four *MRE11A* SNPs) using three genetic models (Models 1–3 detailed in Table S7), we focused on two pairwise interactions with the strongest effects, rs1805812 (*NBS1*) x rs625245 (*MRE11A*) and rs1805812 (*NBS1*) x rs607974 (*MRE11A*). As shown in Table 3, the strongest interaction association was observed for rs1805812 x rs607974 under Model 3, with the strongest effect for heterozygotes or homozygotes for the minor allele on both gene loci (*MRE11A*, *NBS1*). The association observed for rs1805812 x rs625245 also shows the strongest effect under Model 3 (Table 4). Interestingly, both of these interactions replicate independently in both stage 1 and 2.

**Table 3:** Association analysis of rs1805812 x rs607974 interactions between *MRE11A* and *NBS1* with the risk of NHL.

*NBS1* (rs1805812)/			Stage 1				Stage 2			Stage 1 & 2 [Aggregate]		
*MRE11A* (rs607974) genotype			Cases	Controls	OR (95% CI)	Cases		Controls	OR (95% CI)	Cases	Controls	OR (95% CI)
**Model 1**												
	*NBS1* TT/*MRE11A* GG		167	333	1.0 (Ref)	241		362	1.0 (Ref)	408	695	1.0 (Ref)
	*NBS1* TT/*MRE11A* AG,AA; *NBS1* CT,CC/*MRE11A* GG		213	347	1.22 (0.95–1.58)	208		336	0.93 (0.73–1.18)	421	683	1.05 (0.88–1.25)
	*NBS1* CT,CC/*MRE11A* AG,AA		9	38	0.47 (0.22–1.00)	12		42	0.43 (0.22–0.83)	21	80	0.45 (0.27–0.73)
	*P* for interaction		0.8713			0.03153				0.07991		
**Model 2**												
	*NBS1* TT, CT/*MRE11A* GG, AG		354	656	1.0 (Ref)	435		697	1.0 (Ref)	789	1353	1.0 (Ref)
	*NBS1* CC/*MRE11A* GG,AG,AA; *NBS1* TT,CT/*MRE11A* AA		35	62	1.05 (0.68–1.61)	26		43	0.97 (0.59–1.6)	61	105	1 (0.72–1.38)
	*P* for interaction		0.4434			0.6881				0.9218		
**Model 3**												
	*NBS1* TT,CT,CC/*MRE11A* GG; *NBS1* TT/*MRE11A* AG,AA		380	680	1.0 (Ref)	449		698	1.0 (Ref)	829	1378	1.0 (Ref)
	*NBS1* CT,CC/*MRE11A* AG,AA		9	38	0.42 (0.2–0.89)	12		42	0.44 (0.23–0.85)	21	80	0.44 (0.27–0.71)
	*P* for interaction		0.002537			0.002361				0.0000209		

OR = odds ratio; CI = confidence interval.

**Table 4:** Association analysis of rs1805812 x rs625245 interactions between *MRE11A* and *NBS1* with the risk of NHL.

*NBS1* (rs1805812)/		Stage 1			Stage 2			Stage 1 & 2 [Aggregate]		
*MRE11A* (rs625245) genotype		Cases	Controls	OR (95% CI)	Cases	Controls	OR (95% CI)	Cases	Controls	OR (95% CI)
**Model 1**										
	*NBS1* TT/*MRE11A* AA	148	246	1.0 (Ref)	205	278	1.0 (Ref)	353	524	1.0 (Ref)
	*NBS1* TT/*MRE11A* CA,CC and *NBS1* CT,CC/*MRE11A* AA	236	334	1.17 (0.90–1.53)	222	354	0.85 (0.66–1.09)	458	688	0.99 (0.83–1.18)
	*NBS1* CT,CC/*MRE11A* CA,CC	13	46	0.47 (0.25–0.90)	19	48	0.53 (0.31–0.94)	32	94	0.51 (0.33–0.77)
	*P* for interaction	0.4211			0.02209			0.03183		
**Model 2**										
	*NBS1* TT,CT/*MRE11A* AA,CA	367	556	1.0 (Ref)	415	628	1.0 (Ref)	782	1184	1.0 (Ref)
	*NBS1* CC/*MRE11A* AA,CA,CC and *NBS1* TT,CT/*MRE11A* CC	30	70	0.65 (0.42–1.02)	31	52	0.90 (0.57–1.43)	61	122	0.76 (0.55–1.04)
	*P* for interaction	0.03877			0.2747			0.03051		
**Model 3**										
	*NBS1* TT,CT,CC/*MRE11A* AA and *NBS1* TT/*MRE11A* CA,CC	384	580	1.0 (Ref)	427	632	1.0 (Ref)	811	1212	1.0 (Ref)
	*NBS1* CT,CC/*MRE11A* CA,CC	13	46	0.43 (0.23–0.80)	19	48	0.59 (0.34–1.01)	32	94	0.51 (0.34–0.77)
	*P* for interaction	0.00436			0.01478			0.0002523		

OR = odds ratio; CI = confidence interval.

### Imputation and functional predictions using data from the 1,000 Genomes Project

**Figure 1:**
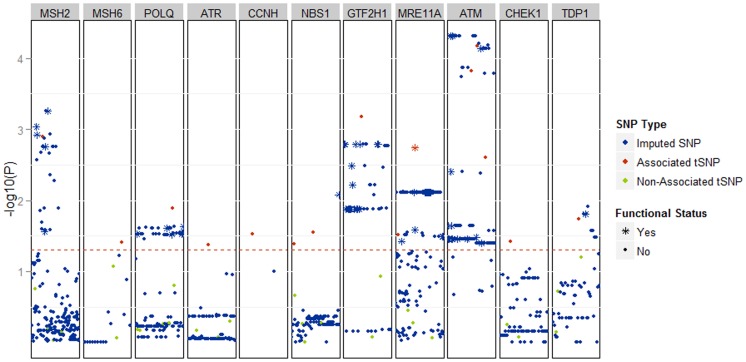
The results of association analysis, displayed as Manhattan plot, after imputation of 28 SNPs genotyped in both stage 1 and 2, which tag 11 DNA repair genes that showed association with NHL risk in our study. The SNPs and genes are ordered by chromosomal position (x-axis). The associations are displayed as –log_10_(p-value) for each SNP. Red dots represent fifteen tagging SNPs that were genotyped in our study and were associated with NHL risk. Green dots represent tagging SNPs that were genotyped in our study and that showed no association with NHL. Blue markers represent SNPs imputed by IMPUTE from 1KG. The red dotted line defines the threshold of p-value <0.05. * indicates an associated SNP with a putatively functional impact; non-synonymous coding change or SNP mapping in: transcription factor binding site, H3K4Me1 chromatin mark, DNaseI hypersensitivity cluster, 5′UTR, 3′UTR.

In order to identify the SNPs with putative functional impact, we imputed all associated loci from the aggregate analysis using data from the 1,000 Genomes Project (1KG; described in Material and Methods). As seen in Figure 1, the association analysis of imputed data did not yield association effects that were stronger than those observed in the analysis of genotyped SNPs. The imputation, however, identified variants that correlate with genotyped SNPs and show an association effect comparable with single SNP analyses of aggregate data (Figure 1). To investigate possible biological implications of these associations we tested the imputed SNPs using ANNOVAR (Materials and Methods). While only one non-synonymous SNP was found among the imputed variants (rs1381057 in *POLQ*), other imputed SNPs map within high-impact regulatory regions. In the *ATM* locus we found numerous putatively functional variants strongly correlated with associated tSNPs (Table 5). Of these, rs228594 merits particular attention; it maps in a DNase I hypersensitivity cluster as well as within *junD* and *FOSL2* binding sites, providing a rationale for future molecular exploration. The detailed list of imputed SNPs with predicted functional impact is in Table S8.

**Table 5:** Putatively functional SNPs imputed from the ATM locus associated with the risk of NHL.

SNP	r^2^ with tSNP*	OR (95% CI)	*P*	phastCons 44-way	TFBS	H3K4me1 chromatin mark	DNase I Hypersensitivity cluster	UTR	Transcribed in GM12878
rs228594	0.991	0.71 (0.60–0.84)	4.85E-05	-	JunD, FOSL2	-	Yes	-	Yes
rs228599	0.991	0.71 (0.60–0.84)	4.85E-05	-	c-Jun	Yes	-	-	Yes
rs227070	0.997	0.71 (0.61–0.84)	7.32E-05	-	-	-	Yes	-	-
rs228595	1	1.16 (1.05–1.29)	0.003922	-	-	Yes	-	-	Yes
rs672655	0.994	0.89 (0.80–0.98)	0.02264	243	-	-	-	-	Yes
rs228596	0.994	0.89 (0.80–0.98)	0.02295	-	-	Yes	-	-	Yes
rs189037	0.994	0.89 (0.80–0.98)	0.02296	-	HEY1, TAF1, IRF4, PAX5-C20, POU2F2	-	-	5′	Yes
rs662578	0.994	0.90 (0.81–0.99)	0.03296	-	-	-	Yes	-	Yes
rs228590	0.994	0.90 (0.81–0.99)	0.03476	-	PAX5-C20	Yes	Yes	-	Yes
rs228598	0.994	0.90 (0.81–0.99)	0.03476	-	c-Jun	Yes	Yes	-	Yes
rs228591	0.994	0.90 (0.81–0.99)	0.03476	-	-	Yes	Yes	-	Yes
rs625120	0.994	0.90 (0.81–0.99)	0.03476	-	-	Yes	-	-	Yes
rs228597	0.994	0.90 (0.81–0.99)	0.03476	-	-	Yes	-	-	Yes
rs228592	0.994	0.90 (0.81–0.99)	0.03476	-	-	-	Yes	-	Yes
rs582157	0.994	0.90 (0.81–0.99)	0.03476	-	-	Yes	-	-	Yes
rs227072	0.998	0.90 (0.81–1.00)	0.04007	-	JunD	-	-	-	Yes
rs425061	0.998	0.90 (0.81–1.00)	0.04007	272	-	-	-	-	Yes
rs227092	0.998	0.90 (0.81–1.00)	0.04007	-	-	-	-	3′	Yes
rs227091	0.998	0.90 (0.81–1.00)	0.04007	-	-	-	-	3′	Yes
rs4585	0.998	0.90 (0.81–1.00)	0.04007	-	-	-	-	3′	Yes

OR = odds ratio; CI = confidence interval; TFBS = transcription factor binding site; UTR = untranslated region.

*Correlated tSNP for rs228595 is rs611646; all other SNPs are correlated with the tSNP rs419716.

### eQTL analysis

**Figure 2:**
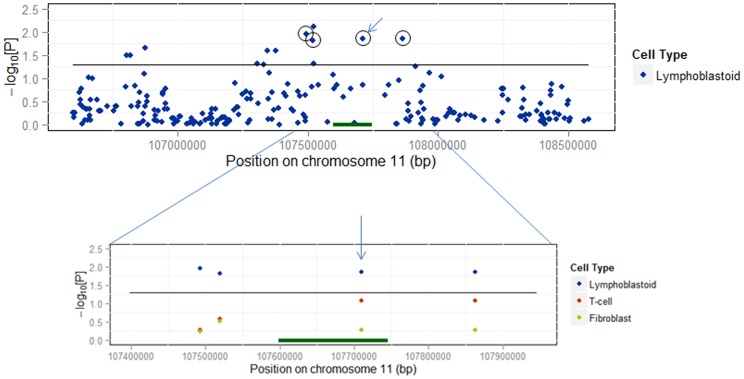
The distribution of eQTLs across the region of *ATM* determined by ILMN_1716231 probe from the data of lymphoblastoid cell lines from 75 individuals of European ancestry. The eQTLs were identified using Genevar. The eQTL association with rs227060 (arrow), the most significant SNP associated with NHL risk in our data, is the second strongest eQTL across *ATM* locus. The circles indicate all SNPs that correlate with rs227060. Zoom in shows eQTL associations of four correlated SNPs in lymphoblastoid cell line versus T-cell and fibroblastoid cell lines. The eQTL associations are displayed as –log_10_(p-value) on y-axis.

Using Genevar [37] we investigated eQTL associations for the *ATM* region by examining publicly available data collected from lymphoblastoid cell lines (LCL), T-cell lines (TCL), and fibroblast cell lines (FCL) derived from 75 individuals of European ancestry [41]. Out of the three *ATM* SNPs associated with NHL risk in our data, the most significant eQTL effect was observed for rs227060 in LCL based on data from probe ILMN_1716231, showing that reduced expression correlates with the risk allele (genetic correlation coefficient rho = 0.284, p = 0.0136; Figure S6). Interestingly, for the probe ILMN_1716231, the association of rs227060 with *ATM* expression was the second most significant eQTL in the region (Figure 2).

## Discussion

Extensive published data suggest that DNA repair pathways are associated with lymphoma susceptibility [26], [27]. The rare syndromes attributed to inherited mutations in DNA repair genes, such as ataxia talangiectasia (A-T; mutations in *ATM*) or Nijmegen breakage syndrome (NBS; mutations in *NBS1*) manifest with early onset lymphomas of various histological subtypes [42], [43]. DNA repair plays a central role in B-cell development in germinal centers of primary and secondary lymphoid organs via V(D)J recombination, which is regulated by the genes involved in double strand break repair, mainly in non-homologous end-joining (NHEJ) [26]. The mouse knockout models of different components of NHEJ have serious defects in the V(D)J recombination process and manifest with increased incidence of B-cell specific lymphomas [26], [44], [45]. Also, inherited immunodeficiency syndromes (e.g. SCID), which are often associated with early onset lymphomas [26] are due to germline mutations in NHEJ genes. This and other evidence points to the importance of DNA repair genes in lymphomagenesis and suggest that these pathways are putative candidates in the susceptibility to lymphoma. In this study we tested common genetic variation in DNA repair genes for its role in susceptibility to NHL. The two-stage design, the thorough computational selection of DNA repair targets, the examination of potential epistatic effects of the associated loci, and the suggested functional implication of associated variants are among the major enhancements of our study design compared to prior efforts.

In this study, we report the most significant association with risk of NHL for two SNPs in the *ATM* locus, remaining significant after adjustment for multiple testing (Bonferroni). Although among FL patients there was no association observed, *ATM* SNPs did correlate with risk of DLBCL and SLL/CLL. While the FL subset was the second largest in the study (n = 301), many other subtypes were underrepresented (n<100) and hence it was not possible to assess risk among these smaller groups due to the limited power (although a suggestive, yet non-significant effect was observed among Mantle Cell lymphomas; rs611646, OR = 1.45, 95% CI: 1.03–2.05, p = 0.035). It is also important to note that the associations of *ATM* SNPs are stronger among the pooled NHL analysis (all subtypes) compared to separate associations with DLBCL or SLL/CLL. This evidence suggests for the first time that *ATM* is a putatively novel candidate NHL susceptibility locus.

In our study we have noted association differences in stratified analyses by AJ status (Table S5). This relates to the two most significant *ATM* SNPs, which show the significant risk effect only in non-AJ population. While the frequency of the risk allele of both SNPs appears to be similar among AJ and non-AJ cases (for rs227060 MAF = 40%, for rs611646 MAF = 48%), the MAF in AJ and non-AJ controls differs by approximately 4% (for rs227060 MAF = 37% and 33% respectively, for rs611646 MAF = 45% and MAF = 42% respectively). While there is a possibility of underlying genetic substructure [46], [47], we believe reduced power of the AJ stratified sub-analysis is the most plausible explanation for the observed differences in the risk effects between AJ and non-AJ subsets, as AJ cases are underrepresented by ∼43% compared to non-AJ cases in the study. It is likely that despite the differences in allele frequency of both SNPs in AJ, the risk would be detected by increasing the number of cases to a comparable size of the non-AJ subset. The issue of power reduction contributing to the observed association differences between AJ and non-AJ is further supported by the same directionality of odds ratios in both AJ and non-AJ subsets and aggregate analysis. To explore both possibilities in detail, a larger validation analysis and possibly the detailed fine mapping of the *ATM* locus in AJ as well as non AJ populations will be needed.

Prior evidence linking *ATM* with lymphoid malignancies has been largely restricted to the somatic level; *ATM* somatic mutations were noted in particular lymphoma subtypes of DLBCL [48], CLL [49]–[52], and MCL [53], [54], consistent with the results of the subtype-specific analysis in our data. Rare germline variants in *ATM* (MAF<0.05) have previously been associated with CLL susceptibility [25], however as the current study focused on common variants (MAF>0.05), these SNPs are not strongly correlated with tSNPs in our study (r^2^<0.1). The study by Sipahimalani, *et al*. performed an extensive analysis exploring the association of six common genetic variants in *ATM* with lymphoma risk [55]. Notably, while the least significant *ATM* SNP associated with NHL risk in our data, rs419716, is strongly correlated with one of the SNPs from that prior study, rs664982 (r^2^>0.9), no association was reported for rs664982 by Sipahimalani, *et al*. [55], although the directionality of the effects for both SNPs is similar. The smaller sample size (798 cases, 793 controls) of this prior report, together with a more heterogeneous population (>15% of Asian ancestry) likely explains the different association outcomes [55]. The different distribution of NHL subtypes in the study by Sipahimalani, *et al*. provides another possible explanation. While our strongest signals appear to be driven by associations of *ATM* SNPs in DLBCL and SLL/CLL, the study population from Sipahimalani, *et al*. had a much higher proportion of FL, for which we did not observe an association in our study. DLBCL specific GWAS studies have been previously reported [7], [12], and most recently a large GWAS by international consortia has identified novel loci in apoptotic pathways associated with the risk of SLL/CLL [11]. While *ATM* was not among the reported associations in these prior studies, these reports focused only on the loci that passed the threshold of genome wide level of significance. For independent confirmation, a separate deeper analysis of this published data will be needed in the follow up study to validate the potential association effect of *ATM* with SLL/CLL and DLBCL in the large populations studied in these scans. Although the replication of our findings in NHL subtypes will be critical as part of the prior and upcoming GWAS studies from large consortia, it will also be very important to test the association effect of identified *ATM* variants in pooled NHL population, where we observed the most significant associations in our analysis. Such rationale is particularly relevant given the critical role of DNA repair pathways (with *ATM* as an important cell cycle checkpoint) in early development of progenitor B and T-cell lineages [56]–[59] via the process of V(D)J recombination producing diverse immune repertoire [60]. Because of the common origin of these precursor cell populations among different NHL subtypes, it is reasonable to hypothesize that variation in DNA repair networks in these progenitor cells could confer risk effects that are shared among multiple NHL subtypes, as we have suggested in our most recent GWAS scan [9] and as also discussed previously [7]. Additionally, double-stranded DNA break and non-homologous end joining repair mechanisms have been implicated in the occurrence of chromothripsis [61], which has recently been observed in both CLL and DLBCL [62]–[64] and proposed along with “chromoplexy” [65] to be involved in a punctuated cancer evolution. *ATM* is a critical checkpoint impacting both homologous recombination and non-homologous end joining [66]. It is possible that the inherited genetic variants in *ATM* may affect the capacity of DSB repair in a way that would associate with the patterns of specific large-scale genomic alterations and rearrangements in a subset of cells in the tumor due to yet unknown genetic or microenvironment modifiers. Although in the context of this study it is highly speculative, given the associations with the *ATM* locus observed here, such a biological scenario is an attractive possibility and should be further investigated in detail on the somatic level of lymphomagenesis.

Numerous other studies have previously examined the germline variation in major DNA repair genes for their association with lymphoma risk [15]–[17], [19]. However, mostly due to the limited selection of candidates, limited power or lack of independent validation, the results were of marginal significance. These borderline associations included the variants in nucleotide excision repair proteins, such as *XRCC1* and *XRCC2* [17], cell cycle protein *BLM* [19], and *MGMT*, a gene involved in DNA repair of alkylation damage [17]. We examined those previously associated loci, which had perfect proxies in our data and observed a borderline association in stage 1 for a proxy of rs1799782 in *XRCC1* [17], (rs3213344, OR = 1.44, 95% CI: 1.03–2.01, p = 0.03), however this SNP did not replicate in our stage 2. *MRE11A* and *NBS1*, which were associated with NHL risk in our study, were examined in a previous report on a small subset of NHL population [18]. Although some marginal associations with NHL risk were observed, the SNPs reported in that previous study were in weak correlation with the variants in *MRE11A* and *NBS1* associated with lymphoma risk in our analysis.

One novelty of our study design compared to prior efforts is the exploratory assessment of potential epistatic effects in DNA repair networks contributing to lymphoma risk. Our data suggest that the interactions between several genetic variants in *MRE11A* and *NBS1*, critical components of the MRN complex, associate with increased risk of NHL. Interestingly, these findings show that the carriers of at least one copy of the minor (protective) allele inherited in both *MRE11A* and *NBS1* are at more than a 2-fold reduced risk of developing NHL compared to the single SNP effect of each locus separately. While these findings are novel they are also exploratory, given the reduced power of this analysis and the fact that none of these observations reached the significance threshold adjusted for multiple testing. Importantly however, these epistatic interactions were observed in both stage 1 and 2 separately (Table 3 and Table 4) suggesting that the observed SNP-SNP associations merit further attention. Looking forward, the addition of other critical checkpoint DNA repair genes (such as *ATM* or *CHEK1/2*) to the MRN interaction model found in our study would be of interest. This analysis, along with the independent validation of epistatic and single SNP associations identified here, will need to be performed in a large consortium as part of the analyses following up on recent reports [11], [67].

Our study provides a suggestive link between the associated SNPs and putatively functional variants to be pursued in subsequent molecular studies. By imputing our data from the public resources of 1KG we have identified several functional variants highly correlated with the SNPs associated with NHL risk in our study. Most notably, several of the variants in *ATM* were located within multiple functional regions, as annotated utilizing ENCODE data from lymphoblastoid cell lines. For example, rs228594, rs228599, and rs189037, which correlate with the associations observed in *ATM*, map within known transcription factor binding sites as well as chromatin marks, DNase I hypersensitivity clusters, and 5′ UTR, strongly supporting a possible impact on expression regulation.

The eQTL association with our top SNP, rs227060, was the second most significant eQTL association within the *ATM* region, and was detected in lymphoblastoid cell lines but not in fibroblast controls [41] (Figure 2). The observed eQTL effect shows the reduced expression of *ATM* correlating with the dosage of rs227060 risk allele. The reduction of *ATM* expression has been strongly linked with radiosensitivity and defective DNA damage-induced *ATM*-dependent signaling in various experimental studies, and was clearly shown to promote the tumor growth in lymphoma and other cancer models [68]. However, despite the potential biological implications of these associations, more detailed molecular investigations will be needed to link the imputed loci with lymphomageneis. Nonetheless, the *in-silico* functional predictions as presented here can substantially improve subsequent fine mapping strategies of associated SNPs. At the same time this approach can reduce the need for a large scale re-sequencing by functional prioritizing the target variants for further molecular investigation.

Besides *ATM*, other loci also showed association effects in pooled NHL or subtype-specific analyses. As power limitations of our study may also be a concern, subsequent validation of these findings should be performed in large consortia. These future studies, with the concomitant collection of epidemiologic data and clinical characteristics, will also allow for a more in-depth analysis of potential gene-environment interactions attributed to DNA repair pathways. At the same time, the utilization of data from completed and ongoing lymphoma GWAS will be critical for the assessment of other interacting molecular pathways that may define the complex genetic susceptibility to NHL. For example, the HLA locus has been consistently replicated as a low-penetrant allele in recent NHL GWAS and follow-up meta-analyses [7], [8], [10], [11], [32], [69]. It has been shown that the innate immunity pathways are closely connected with particular DNA repair networks (e.g. B-cell maturation in germinal centers) [58], [70], suggesting that detailed exploration of such interactions will be important. Our results strongly support the strategies for the pathway analysis of data from current and future GWAS on lymphoma susceptibility, using a deep validation of associations, considering the loci that did not reach genome-wide association thresholds, but may be biologically related. Our observations indicate that the genetic variants in key biological pathways, such as DNA repair, may account for an additional fraction of missing inherited susceptibility to lymphoid neoplasia. The associations observed here can also serve as the basis for further molecular investigations of the biological roles of the implicated loci on both a germline and somatic level. Such investigations will contribute not only to more efficient risk algorithms, but will lead to the improved understanding of lymphomagenesis for more effective targeting of therapy and prevention.

## Supporting Information

Figure S1 **Strategy schema for the selection of candidate DNA repair genes in the study.**
(TIF)

Figure S2 **Quantile/Quantile (Q/Q) plot comparing –log_10_(expected p-value) vs. –log_10_(observed p-value) under different quality metric scores (info).** Based on imputation analysis of 109 genotyped SNPs using IMPUTE2. Q/Q analysis of imputed data was used to select quality score threshold with least inflation of significant SNPs, which we have set at 0.7.
(TIF)

Figure S3 **Quantile/Quantile (Q/Q) plot of the association results from stage 1 SNPs genotyped in 650 NHL cases and 965 controls; −log_10_(expected p-value) vs. −log_10_(observed p-value).** The blue dashded line indicates the inflation factor based on 90% of the least significant SNPs (λ = 1.07).
(TIF)

Figure S4 **LD structure generated by Haploview shown for** ***ATM*** **gene region.** The triangle plot displays correlation between the three tagging SNPs genotyped in the study (r^2^ values). The associations of individual SNPs are displayed as −log_10_(p-value) for each SNP from the main effect aggregate analysis.
(TIF)

Figure S5 **Minor allele frequency plot for non-AJ vs. AJ samples.** Data based on allele frequencies in the aggregate analysis (for 109 SNPs with genotype information in stage 1 and stage 2). Blue dots indicate those SNPs associated with NHL in the aggregate analysis. Only 15 SNPs (14%) showed a difference in MAF >0.05. Indicated by arrows are NHL associated SNPs which did show a MAF difference >0.05 between non-AJ and AJ samples.
(TIF)

Figure S6 **Genotype vs. expression (eQTL) results for rs227060 and four expression probes across the** ***ATM*** **locus.** Data generated from lymphoblastoid cell lines (GenCord-L), T-cell lines (GenCord-T) and fibroblastoid cell lines (GenCord-F) established from 5 individuals of European ancestry. The most significant association for rs227060 in GenCord-L has been observed for ILMN-1716231. Data were generated using Genevar.
(TIF)

Table S1 **Summary of the demographic characteristics of the case/control study population.**
(XLSX)

Table S2 **List of DNA repair genes and tagging SNPs genotyped.** Chromosome and base pair position are based on GRCh37/hg19 build. Allele frequencies calculated among our sample population.
(XLSX)

Table S3 **Results of single SNP associations analysis of tSNPs in DNA repair genes with the risk of NHL observed in our study, including 109 SNPs genotyped in stage 1 and stage 2.**
(XLSX)

Table S4 **Full list of haplotype associations (p<0.05) for DNA repair genes associated with NHL in our study.**
(XLSX)

Table S5 **Stratified AJ and non-AJ results for SNPs associated with NHL in the main effect aggregate analysis.**
(XLSX)

Table S6 **Results from pairwise SNP-SNP interaction associations of tSNPs in DNA repair genes with NHL risk.** A) Pairwise comparisons among the 15 tSNPs associated with NHL risk in the aggregate main effect analysis. B) Pairwise comparisons of 15 tSNPs with all additional SNPs genotyped in both stage 1 and stage 2 (n = 94). Associations between *MRE11A* and *NBS1* are shaded in grey.
(XLSX)

Table S7 **Pairwise interaction results between** ***NBS1*** **(rs1805812) and** ***MRE11A*** **(rs607974, rs625245, rs557148, rs10831227) and NHL risk under three different genetic models.** Each model was tested for a different SNP x SNP combination between rs1805812 and 4 SNPs tagging *MRE11A*. Indicated are the different combinations of genotypes used for each respective genetic model.
(XLSX)

Table S8 **Summary of tagging and imputed SNPs in DNA repair genes associated with NHL risk with high predicted functional impact.** Functional information was annotated using ANNOVAR, and included data generated from the ENCODE project.
(XLSX)
