# Supplementary figures and images for: Genetic Variation in DNA Repair Pathways and Risk of Non-Hodgkin's Lymphoma
Source: PLoS One. 2014 Jul 10;9(7):e101685. doi: 10.1371/journal.pone.0101685 (PMC4092067; doi:10.1371/journal.pone.0101685)

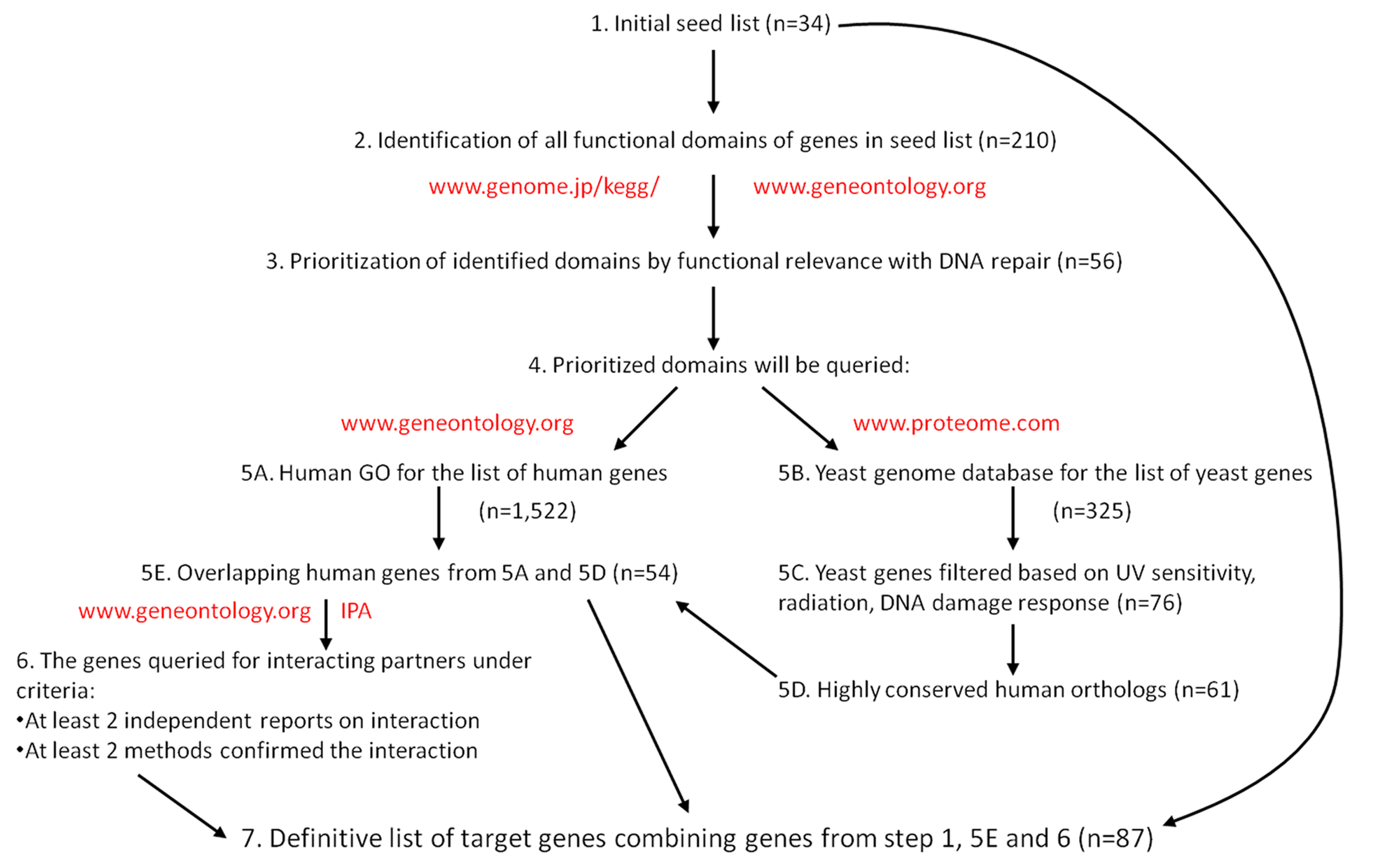

Supplement: Figure S1 — Strategy schema for the selection of candidate DNA repair genes in the study. (TIF) [file pone.0101685.s001.tif]

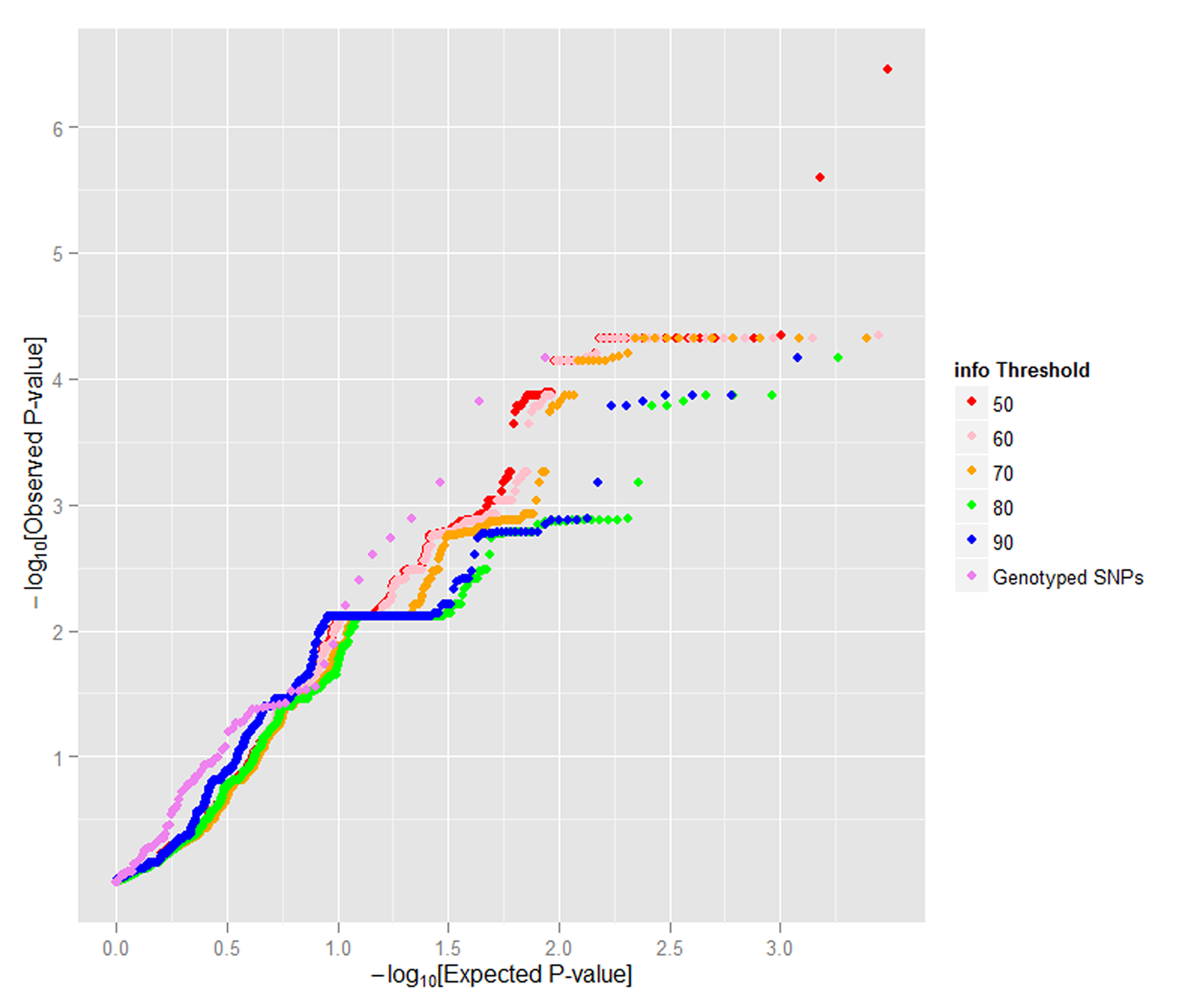

Supplement: Figure S2 — Quantile/Quantile (Q/Q) plot comparing –log10(expected p-value) vs. –log10(observed p-value) under different quality metric scores (info). Based on imputation analysis of 109 genotyped SNPs using IMPUTE2. Q/Q analysis of imputed data was used to select quality score threshold with least inflation of significant SNPs, which we have set at 0.7. (TIF) [file pone.0101685.s002.tif]

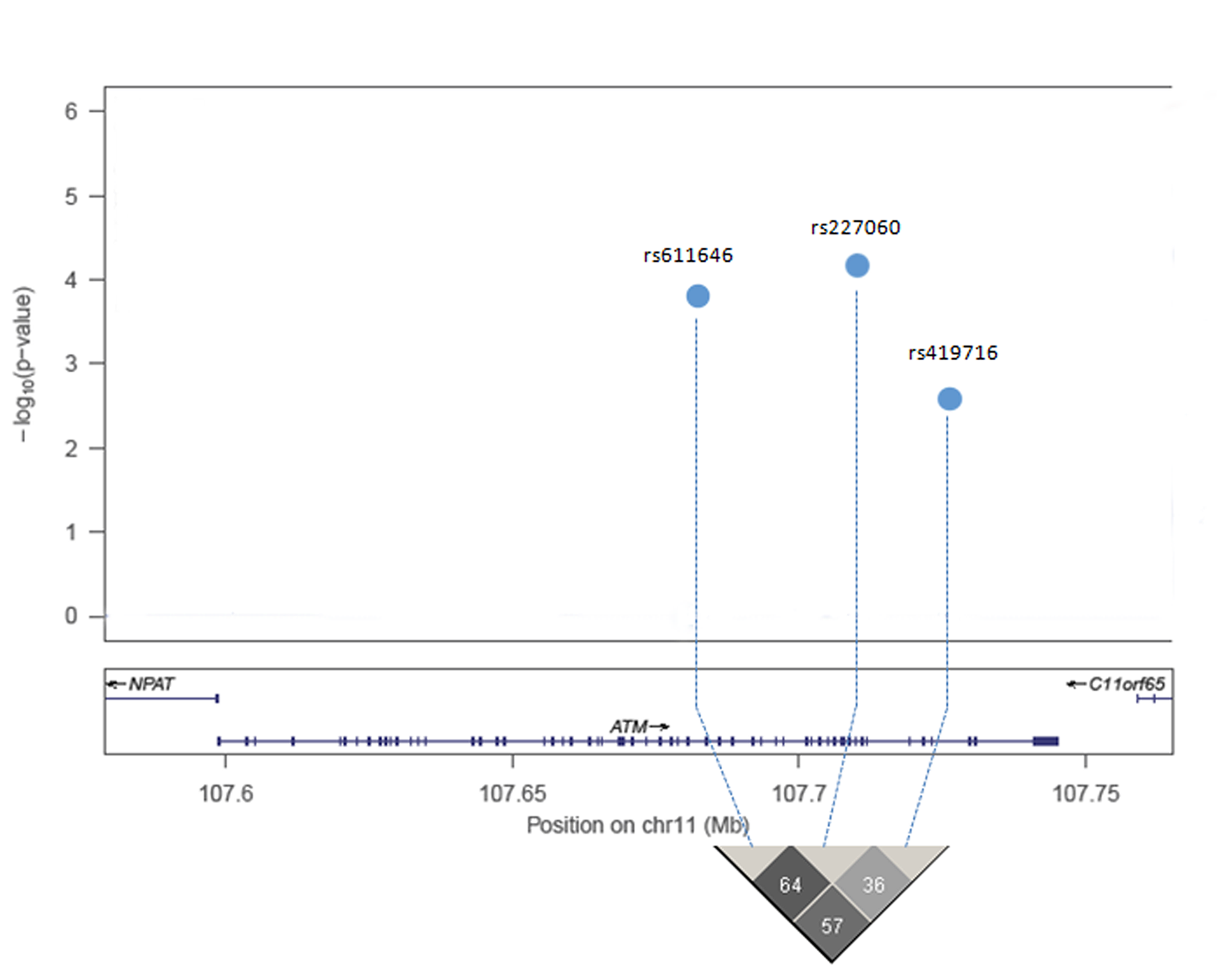

Supplement: Figure S4 — LD structure generated by Haploview shown for ATM gene region. The triangle plot displays correlation between the three tagging SNPs genotyped in the study (r2 values). The associations of individual SNPs are displayed as −log10(p-value) for each SNP from the main effect aggregate analysis. (TIF) [file pone.0101685.s004.tif]

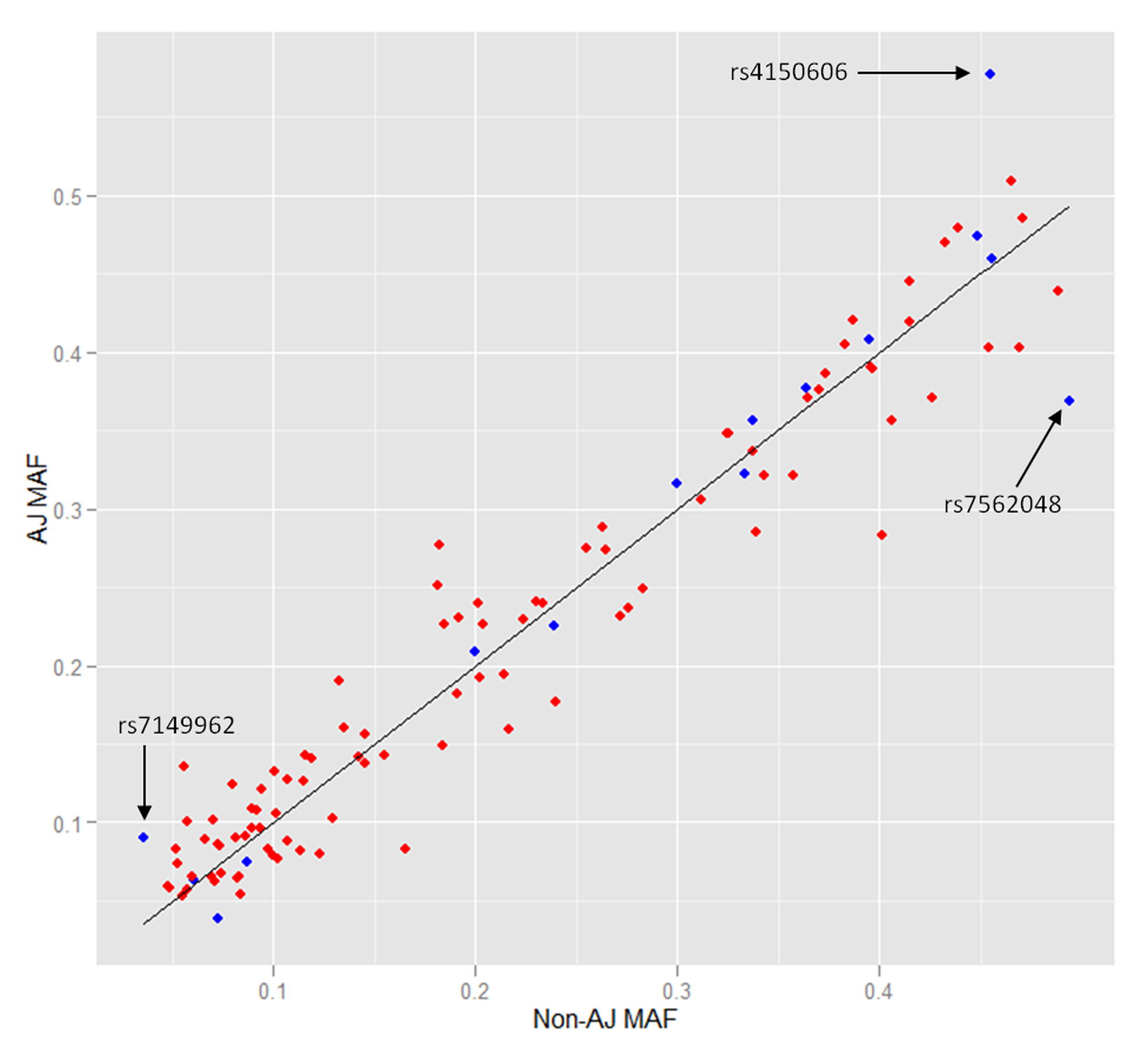

Supplement: Figure S5 — Minor allele frequency plot for non-AJ vs. AJ samples. Data based on allele frequencies in the aggregate analysis (for 109 SNPs with genotype information in stage 1 and stage 2). Blue dots indicate those SNPs associated with NHL in the aggregate analysis. Only 15 SNPs (14%) showed a difference in MAF >0.05. Indicated by arrows are NHL associated SNPs which did show a MAF difference >0.05 between non-AJ and AJ samples. (TIF) [file pone.0101685.s005.tif]

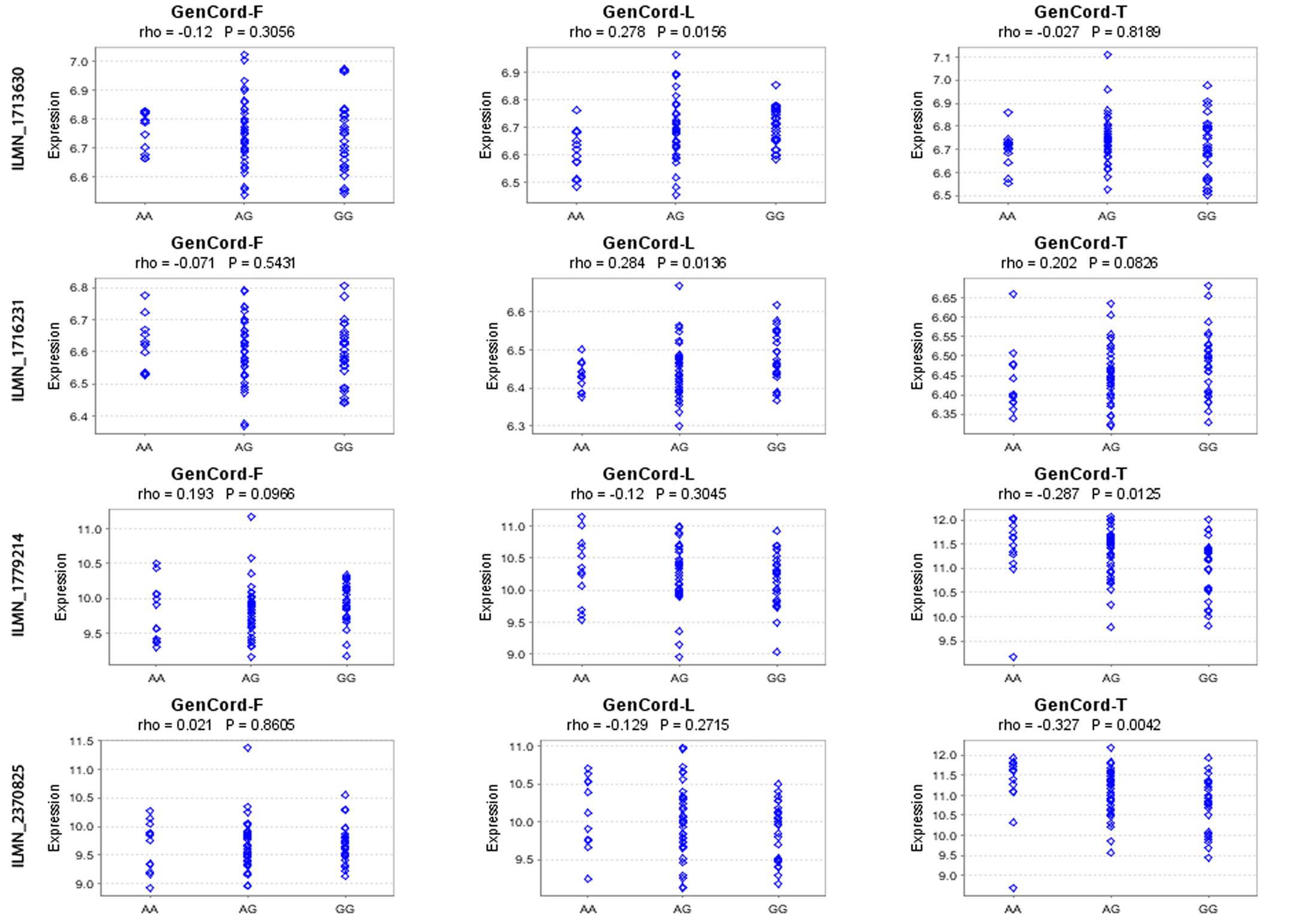

Supplement: Figure S6 — Genotype vs. expression (eQTL) results for rs227060 and four expression probes across the ATM locus. Data generated from lymphoblastoid cell lines (GenCord-L), T-cell lines (GenCord-T) and fibroblastoid cell lines (GenCord-F) established from 5 individuals of European ancestry. The most significant association for rs227060 in GenCord-L has been observed for ILMN-1716231. Data were generated using Genevar. (TIF) [file pone.0101685.s006.tif]
